# AngioVac thrombectomy in patient with right atrial thrombus and gastrointestinal bleed: case and literature review

**DOI:** 10.1093/omcr/omaa138

**Published:** 2021-02-15

**Authors:** Hernán L Vera-Sarmiento, David Hurtado-de-Mendoza, Rosario Colombo

**Affiliations:** 1 School of Medicine, Universidad Industrial de Santander, Bucaramanga, Santander, Colombia; 2 Department of Internal Medicine, Jackson Memorial Health System. University of Miami Hospital. Miami, Florida, USA; 3 Department of Cardiology, Jackson Memorial Hospital, Miami, USA

## Abstract

Catheter-directed thrombectomy is a novel promising therapy with little published experience. Previous reports have described it as a useful tool in high risk patients in need of intravascular material resection. Here we present a unique and never reported case of AngioVac device thrombectomy use in a patient with right atrial catheter-associated thrombus and gastrointestinal bleed that contraindicated other thrombectomy therapies due to severe anemia and high bleeding risk. A brief literature review about this therapy and its main outcomes is presented to contextualize the reader and contribute to academic knowledge.

## INTRODUCTION

Right heart masses are considered a life-threatening condition due to a high risk of pulmonary embolism (PE). Masses found to be thrombi are rare and have various etiologies such as thrombosed central veins or central venous catheter-associated masses [1]. Non-thrombotic causes are usually related to metastatic lesions from tumors of the liver, lungs, mediastinum and melanoma [2].

**Figure 1:**
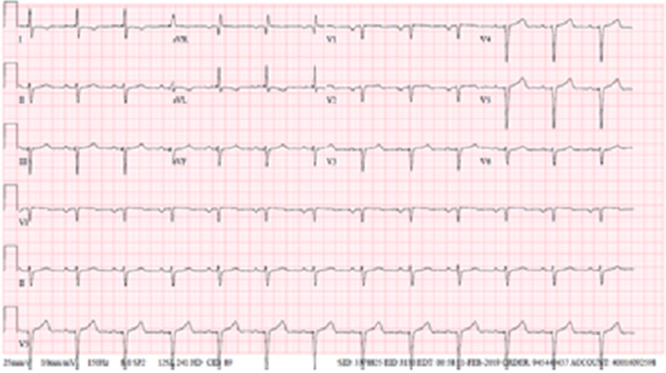
Admission electrocardiogram.

Diagnosis is based on transesophageal echocardiogram (TEE), however its sensibility and specificity for differentiating between clots, myxomas, fibromas and fatty tissue is limited, hence, additional imaging is usually required, such as contrast perfusion transthoracic echocardiogram (TTE) or TEE. magnetic resonance imaging (MRI) and computerized tomography (CT) scan have similar sensitivity but greater specificity compared to TTE at defining the etiology of the cardiac mass and may be useful for differentiating between malignant, vascular and stromal tumors from thrombi [3].

Once the diagnosis is made, treatment is rapidly targeted with different therapies as pharmacological thrombolysis, surgical mechanical thrombectomy and mechanical catheter-directed therapies as the AngioVac device. Choosing among these methods is still debated, retrospective studies have shown that patients with PE and free floating intra-cardiac thrombus benefit from surgical thrombectomy as opposed to thrombolysis alone. In those cases where surgery is contraindicated, thrombolytic therapy alone is an option if there is not a high bleeding or distal embolization risk [2].

Patients who are not eligible for those therapies or have a large clot burden are candidates for catheter thrombectomy with AngioVAC device, a novel promising therapy but with little available published experience and evidence. Here we present the case of a patient with right atrial catheter-associated thrombus and upper gastrointestinal bleeding (UGB) treated with AngioVac device. A brief literature review has been included for knowledge contribution to the reader.

## CASE REPORT

A 29-year-old Guatemalan male with significant past medical history of end-stage renal disease on intermittent hemodialysis, hypertension and 5 years use of indwelling tunneled central venous catheter presented to the emergency room due to a 5-day clinical onset of black stools and 1 day of acute moderate chest pain, palpitations, shortness of breath at rest and dizziness. Vital signs were only remarkable for a blood pressure of 182/103 mmHg. At physical examination head, neck, lungs, heart and abdomen were normal. No blood per rectum or melena was found. Bilateral 2+ lower extremity pitting edema was noted.

Laboratory reports showed positive Troponin-I consecutive results of 4.58—4.56—4.19 ng/L and severe acute anemia with Hemoglobin (HgB) of 5.4 g/dl, EKG showed sinus rhythm, left atrial enlargement, left anterior fascicular block, poor R wave progression and no ST-segment changes (see Fig. 1). TTE was ordered. Blood cultures were positive for *Staphylococcus epidermidis*. The patient’s chart showed last HgB level of 10.4 g/l 15 days before in prior admission.

**Figure 2:**
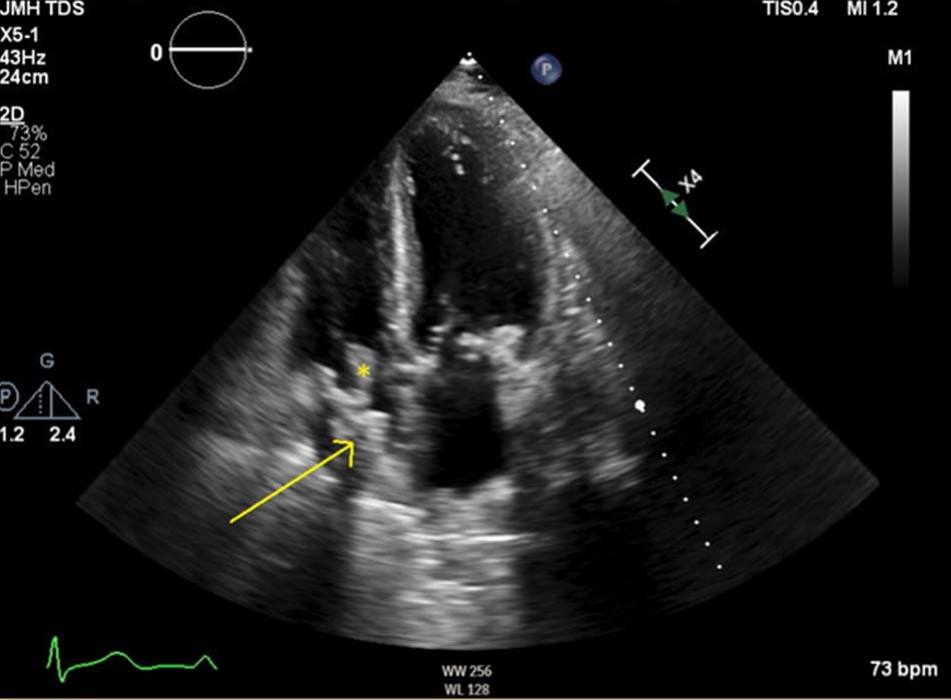
Apical four-chamber view showing thrombus in right atrium (arrow) with mobile element (asterisk).

Patient was diagnosed with acute UGB and type II non-ST Elevation myocardial infarction (NSTEMI) secondary to acute HgB drop and catheter related sepsis (six points by SOFA); he was subsequently started on pantoprazole drip, beta blocker, rosuvastatin and three packed red blood cells (pRBC) transfusions were given. Anti-platelet agents and anticoagulants were held because of UGB and pneumatic compression was ordered. Hemodialysis continued as usual and erythropoietin was administered. Vancomycin was started for treatment of *S. epidermidis* bacteremia and endoscopy showed no bleeding, erythema or inflammatory changes suggestive of gastritis or ulceration in the entire examined stomach.

**Figure 3:**
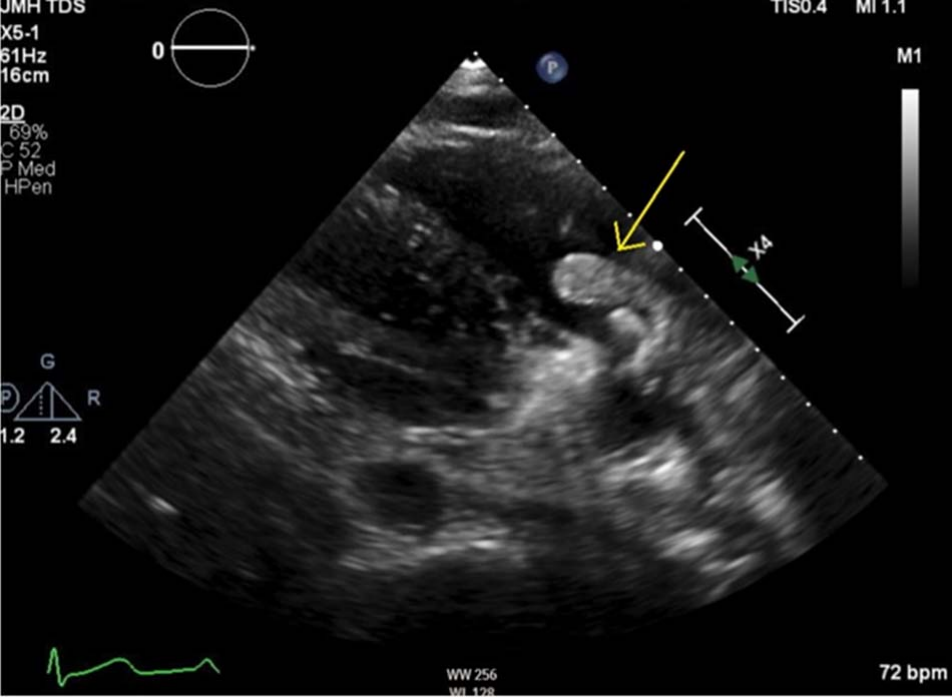
Parasternal right ventricular inflow view showing mobile thrombus (arrow).

TTE demonstrated normal LVEF with a highly mobile, irregularly shaped echodensity (2.7 cm in maximum diameter) inside the right atrium (RA), suggestive of a catheter-associated thrombus (see Figs. 2 and 3). Due to high risk of embolization and sudden death risk he was admitted to intensive care unit (ICU) and cardio-thoracic surgery (CTS) was consulted.

CTS performed percutaneous removal under general anesthesia with Angiovac device because of bleeding risk of open thrombectomy. Intra-operative TEE showed no residual valve abnormalities and no remaining thrombus. After procedure, there were no immediate complications and patient remained in Surgical ICU for monitoring; 1 day later patient was extubated uneventfully. Pathology revealed calcified thrombus and negative culture growth. Patient was started on heparin anticoagulation on post-op Day 2, which was later bridged to warfarin 5 mg daily. He was then transferred to medical floor on post-op Day 3 and started ambulating without difficulties. HgB level increased to 8.3 g/dl following one more transfusion of pRBC. Patient was then discharged following several persistently negative blood cultures to complete 2-week course of outpatient vancomycin. Catheter was replaced for a right femoral vein one and patient continued with previous three times a week dialysis regimen.

## DISCUSSION

Introduced in 2009, the AngioVac device is a vacuum-assisted thrombectomy system designed for *en bloc* removal of large clot burden while avoiding bleeding and hemolysis risks associated with fibrinolytics, surgery and rheolytic systems [4, 5]. Here we present a case of AngioVac use considered clinically relevant as this appears to be the first case of its use in a patient with an acute contraindication to anticoagulation.

In our literature review, a total of 45 cases of AngioVac use for thrombus aspiration was obtained including 15 cases of a single center case reports published by Donaldson *et al*. [11] in 2015. In those 45 cases, main clinical manifestation was dyspnea in 13 cases, followed by incidental finding of the thrombi in six cases and palpitations in five, which is consistent with this presented case. The initial diagnosis was made by TEE in seven cases, not specified in nine cases, CT scan and venogram in seven and four cases, respectively. Only three cases were initially diagnosed by TTE as the one reported here [6–11]. In 15 cases, neither the clinical manifestations nor diagnostic approach was described. No report described UGB as a clinical finding.

The obtained material was removed from the inferior vena cava (IVC) in more than half of the cases, 11 of these cases had extent to RA. Only eight cases were isolated RA thrombi of which just six were related to catheters, consistent with epidemiologic data about rarity of RA thrombosis and highlighting the importance of the presented case. Obtained material consisted of thrombi in 80% of the cases, the remaining was associated to malignancy or vegetation. Only four cases reported a failed procedure and complications were reported in six cases with one septic emboli case and hematomas for the remaining five. [6–11]. Particularly, in the Donaldson *et al*. [11] case report, 11 cases had post-procedural bleeding of which five needed transfusions due to overt bleeding, and an overall 87% of survival to discharge was reported, which suggests the AngioVac thrombectomy as an overall safe procedure for those high surgical risk patients.

A recent meta-analysis by Hameed *et al*. [12] was presented in 2019 with promising outcomes of this therapy. In this study, a total of 182 cases of AngioVac use were analyzed, of which 101 corresponded to thrombus removal. Successful removal pooled rate was 80.5 and 32.4 for right atrium/inferior vena cava (RA/IVC) thrombi and PE, respectively. As similar to the previously described cases, the need of conversion to open surgery had a low rate, with 12.3 pooled rate for thrombi removal. However operative mortality pooled rate was 14.8 and 32.3 for RA/IVC thrombi and PE, respectively, which should be taken into consideration when treating PE patients [12].

All this being said, percutaneous thrombectomy with AngioVac device is a promising therapy, which needs more complex trials to evaluate specific outcomes and to answer questions such as precise indications and contraindications of this intervention as well as complication and failure rates. Available reports and recent meta-analysis suggest AngioVac thrombectomy as a viable option for those high surgical risk patients with an inspiring high successful and low mortality rates except for those patients with PE, in which outcomes seem to be worse with this therapy.
